# Identification of Tyrosine-9 of MAVS as Critical Target for Inducible Phosphorylation That Determines Activation

**DOI:** 10.1371/journal.pone.0041687

**Published:** 2012-07-26

**Authors:** Chaoyang Wen, Zhifeng Yan, Xiaoli Yang, Kai Guan, Changzhi Xu, Ting Song, Zirui Zheng, Wenjun Wang, Ying Wang, Man Zhao, Yanhong Zhang, Tao Xu, Jianping Dou, Jingmei Liu, Quanbin Xu, Xiang He, Congwen Wei, Hui Zhong

**Affiliations:** 1 Hainan Branch of Chinese PLA General Hospital Sanya City, Hainan, China; 2 Beijing Institute of Biotechnology, Beijing, China; 3 The General Hospital of People's Armed Police Hospital, Beijing, China; 4 Beijing Institute of Disease Control and Prevention, Beijing, China; University of Montreal, Canada

## Abstract

**Background:**

Innate immunity to viruses involves receptors such as RIG-I, which senses viral RNA and triggers an IFN-β signaling pathway involving the outer mitochondrial membrane protein MAVS. However, the functional status of MAVS phosphorylation remains elusive.

**Methodology/Principal Findings:**

Here we demonstrate for the first time that MAVS undergoes extensive tyrosine phosphorylation upon viral infection, indicating that MAVS phosphorylation might play an important role in MAVS function. A tyrosine-scanning mutational analysis revealed that MAVS tyrosine-9 (Y9) is a phosphorylation site that is required for IFN-β signaling. Indeed, MAVS Y9F mutation severely impaired TRAF3/TRAF6 recruitment and displayed decreased tyrosine phosphorylation in response to VSV infection compared to wild type MAVS. Functionally, MAVS Y9 phosphorylation contributed to MAVS antiviral function without interfering with its apoptosis property.

**Conclusions/Significance:**

These experiments identify a novel residue of MAVS that is crucially involved in the recruitment of TRAF3/TRAF6 and in downstream propagation of MAVS signaling.

## Introduction

 The innate immune system, which is characterized by the production of type-1 interferon (IFN)-α/β cytokines and by the activation of natural killer (NK) cells, plays important roles in invading pathogens detection and elimination [1], [2]. In most cell types, RIG-I (retinoic acid inducible gene I) and MDA-5 (melanoma differentiation-associated gene 5) are thought to be the primary receptors that transduce signals using a common intermediary protein called MAVS (mitochondrial antiviral signaling; also known as IPS-1/VISA/Cardif) to stimulate IFN-α/β production in response to infections by RNA viruses [3], [4], [5], [6]. Both RIG-I and MDA5 contain caspase activation and recruitment domains (CARDs) that interact with the CARD domain-containing protein MAVS upon binding to uncapped dsRNA. Binding of RIG-I and MDA-5 to MAVS through CARD-CARD homotypic interactions leads in some way to the stabilization and accumulation of MAVS homodimers. These homodimers are able to directly recruit ubiquitin ligase TRAF3 (tumor necrosis factor receptor-associated factor 3) and to activate its E3 ligase activity. Active TRAF3, in turn, may mediate the recruitment and activation of TBK1 through scaffold molecules, such as NEMO (NF-κB essential modulator) and TANK (TRAF family member-associated NF-κB activator) [7], [8].

The MAVS molecule has emerged as the key regulatory in RIG-I signaling, signal transduction downstream of MAVS culminates in the activation of the IRF3/7 (interferon regulatory factors 3 and 7) and NF-κB transcription factors [9]. Posttranslational modifications such as ubiquitination and phosphorylation also play an essential role in the regulation of this complex activation cascade. For example, K63-linked ubiquitination of proteins like RIG-I and NEMO promotes protein-protein interaction and pathway activation, whereas K48-linked ubiquitination, as in the case of IRF-3 and RIG-I, leads to proteasomal degradation and shutdown of signaling [10], [11], [12], [13]. Recent studies showed K63-linked ubiquitination of MAVS following viral infection modulates interaction with key components of the RIG-I signaling [14]. In the present study, we demonstrate for the first time that MAVS undergoes extensive tyrosine phosphorylation upon viral infection. A tyrosine-scanning mutational analysis reveals that MAVS Y9F is strongly impaired in the recruitment of TRAF3 and TRAF6. These findings identify a novel residue of MAVS crucially involved in the formation of higher order complexes containing TRAF3 and required for the propagation of the RIG-I signaling.

## Materials and Methods

### Cell Culture and Transfection

HEK293T, MCF-7 and HepG2 (ATCC; Manassas, VA) cells were grown in Dulbecco's modified Eagle's medium (Invitrogen, Carlsbad, CA) supplemented with 10% heat-inactivated fetal bovine serum (Hyclone), 2 mM L-glutamine, 100 U/ml penicillin, and 100 mg/ml streptomycin. Vectors and Epitope Tagging of Flag-tagged MAVS, Myc-tagged MAVS, Myc-tagged TRAF3, Myc-tagged TRAF6 and Myc-tagged STING were expressed by cloning the genes into the pcDNA3-based vector (Invitrogen). Flag-MAVS mutation plasmids were made based on the pcDNA3-Flag-MAVS plasmid using Quick-Change Mutagenesis kit (Invitrogen). Transient transfections were performed with Lipofectamine 2000 (Invitrogen) according to the manufacturer's instructions.

MAVS−/− mice on a 129/Sv/C57BL/6 background were a kind gift from Dr. Zhijian. Chen [15]. Embryonic fibroblasts (MEFs) from wild type (WT) and mutant mice were prepared from day 15 embryos and cultured in DMEM supplemented with 10% fetal bovine serum.

### Immunoprecipitation and Immunoblotting Analysis

Cell lysates were prepared in lysis buffer (50 mM Tris-HCl [pH 7.5],1 mM phenylmethylsulfonyl fluoride, 1 mM dithiothreitol, 10 mM sodium fluoride, 10 mg/ml aprotinin, 10 mg/ml leupeptin, and 10 mg/ml pepstatin A) containing 1% Nonidet P-40. Soluble proteins were subjected to immunoprecipitation with anti-Flag (M2, Sigma, St Louis, MI), anti-Myc (Santa Cruz Biotech, Santa Cruz, CA), or anti-mouse IgG antibody (Sigma). An aliquot of the total lysates (5%, v/v) was included as control. Immunoblotting analysis was performed with anti-Myc, HRP-conjugated anti-Flag (Sigma), anti-β-Tubulin (Sigma) and anti-MAVS (Cell Signaling Technology, Danvers, MA). The antigen-antibody complexes were visualized by chemiluminescence (Perkin Elmer Life Sciences Inc., Boston, MA).

### Luciferase Reporter Assays

HEK293T cells were transfected with 0.2 µg of the Luciferase reporter pNF-κB-LUC or IFN-β-LUC plus 0.02 µg of the internal control reporter pRL-TK, with or without MAVS expression vector. Transfected cells were collected and Luciferase activity was assessed. All experiments were repeated at least three times in duplicate.

### Immunofluorescence Assay

Cells were washed briefly in PBS, fixed in 4% paraformaldehyde (PFA) in PBS for 10 min, and the nuclei were stained for 10 min with DAPI. After a final washing in PBS, samples were preserved in glycerol and images were captured using a digital camera under a confocal microscope (Zeiss LSM510).

### Subcellular Fractionation

Cells were harvested in low-salt buffer [10 mM Hepes (pH 7.9), 10 mM KCl, 0.1 mM EDTA, 0.1 mM EGTA, 50 mM NaF, 1.5 mM Na_3_VO_4_, protease inhibitor mixture (Roche), 1 mM DTT, 1 mM PMSF] and passed through a 25G needle 15 times. Nuclear fractions were collected by centrifugation at 1,000×g for 5 min at 4°C. Mitochondrial fractions were collected by further centrifugation of the supernatants at 10,000×g for 5 min at 4°C. The supernatants were further fractionated by centrifugation at 100,000×g for 30 min at 4°C to collect membrane and cytosolic fractions.

### RNA Analysis

First-strand cDNA was generated from total RNA using random priming and Moloney murine leukemia virus (M-MLV) reverse transcriptase (Invitrogen). Real-time PCR was performed using QuantiTect SYBR Green PCR Master Mix (Qiagen, Valencia, CA) in triplicate experiments and analyzed on an ABI Prism 7700 analyzer (Applied Biosystems, Foster City, CA). All real-time values were normalized to 18S ribosomal RNA. IFN-β using the following primers:

IFN-β S, 5 -CACGACAGCTCTTTCCATGA-3;

IFN-β AS, 5 -AGCCAGTGCTCGATGAATCT-3.

### Viral Infections

Cells were plated in 10 cm plates at a density of 1×10^6^ cells/plate and incubated overnight. Viral infection was performed when 60% cell confluence was reached. Culture media was replaced by serum-free DMEM, and VSV was added into the media at multiplicity of infection (MOI) as indicated, respectively. After 1 h incubation, extracellular virus was removed by washing cells twice with serum-containing media. Cells and supernatant were harvested at indicated times post-infection.

### Cell Viability Assays

For Trypan blue exclusion test of cell viability, 10 ml of cell suspension was mixed with 10 ml of 0.4% trypan blue solution (Sigma), and both unstained (viable) and stained (dead) cells were counted on the hemacytometer. The percentage of dead cells was calculated by dividing the number of stained cells by the total number of cells.

### Flow Cytometry

For propidium iodide staining, cells were harvested by trypsinization, fixed with ice-cold 70% ethanol and resuspended in a solution containing 50 µg/mL propidium iodide, 0.1% Triton X-100, 50 µg/mL RNase A, and 5 mM EDTA at room temperature for 1 h. Cells were then diluted 1∶1 in 1% BSA PBS for cytometric analysis. DNA content was assessed by staining 70% ethanol-fixed cells with propidium iodide and monitoring by FACScan (BD Bioscience, San Diego, CA). Annexin V staining was performed as indicated with the Annexin V-Biotin Apoptosis Detection Kit (BD Bioscience). Briefly, cells were washed with cold PBS, resuspended in 1× Binding Buffer and then Annexin V Dye was added, after 15 min incubation, cells were washed and propidium iodide was added, cells were analyzed by flow cytometry. The results were expressed as the mean of three independent experiments.

### Statistical analysis

Analyses were done using the statistical software SAS/STAT. Data analyses over time were undertaken by repeated measures analysis using SAS/STAT. P<0.001 was considered the threshold value for statistical significant significance.

## Results

### MAVS undergoes tyrosine phosphorylation in response to virus infection

**Figure 1:**
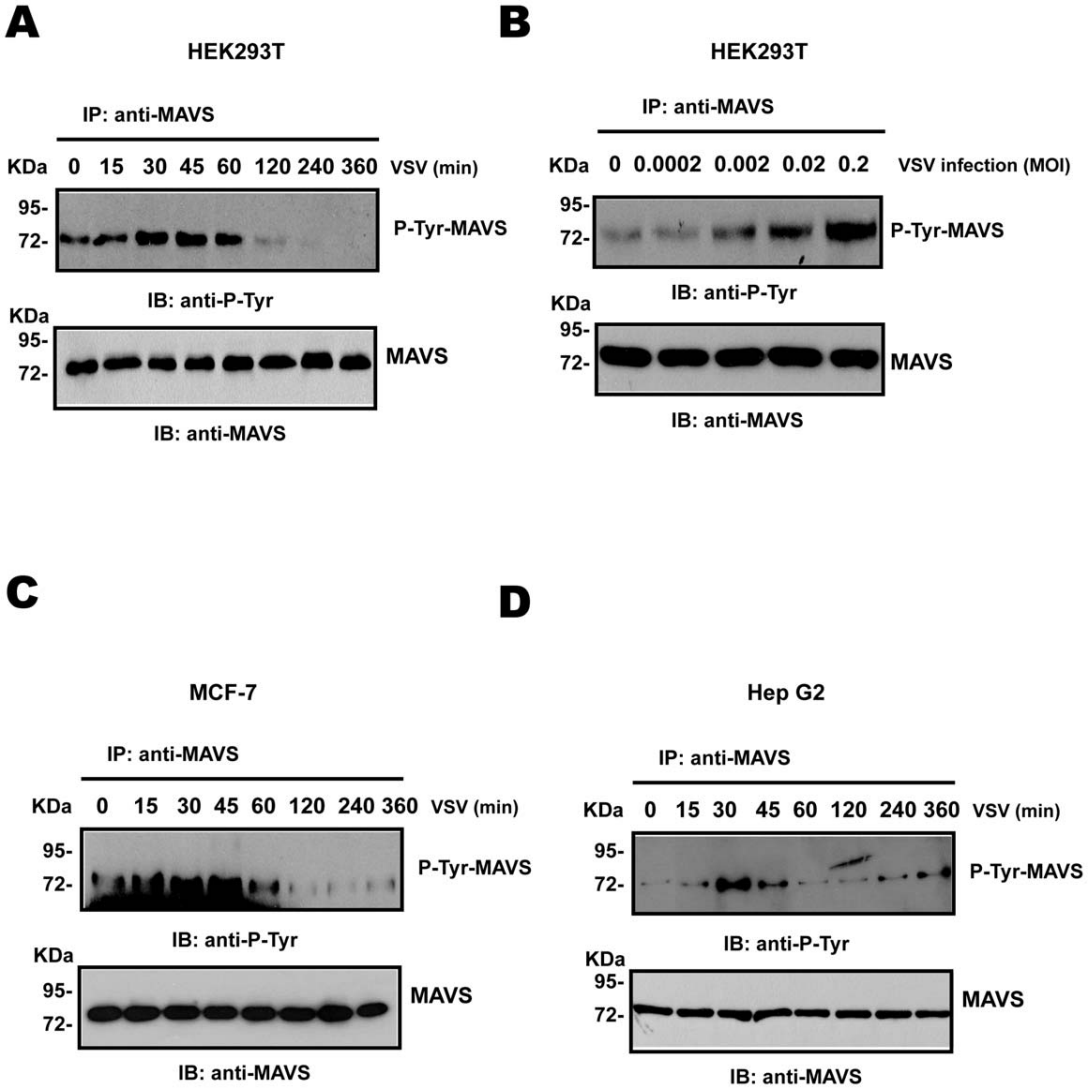
MAVS undergoes tyrosine phosphorylation in response to virus infection. (A–B) HEK293T cells were infected with VSV at a multiplicity of infection (MOI) of 0.002 for the indicated times (A) or at a different MOI for 30 min (B). Anti-MAVS immunoprecipitates were analyzed by immunoblotting with anti-P-Tyr or anti-MAVS antibody. (C–D) MCF-7 (C) or HepG2 (D) cells were infected with VSV at a MOI of 0.002 for the indicated times. Anti-MAVS immunoprecipitates were analyzed by immunoblotting with anti-P-Tyr or anti-MAVS antibody.

Recent studies showed that ubiquitination at MAVS K500 mediates recruitment of IKKε to MAVS, leading to MAVS phosphorylation and negative regulation of the NF-κB activation, raising the possibility that phosphorylation may be involved in this process [14]. To this end, endogenous MAVS was immunoprecipitated with anti-MAVS antibody at different times after VSV infection, and phosphorylation was detected with a tyrosine phosphotyrosine-specific antibody. Fig. 1A demonstrates that MAVS phosphorylation occurred as early as 15 min post-infection and increased between 30 min and 60 min. In concert with this finding, MAVS also underwent tyrosine phosphorylation as virus titers increased (Fig. 1B). Furthermore, MAVS phosphorylation also occurred in human breast cancer MCF-7 cells and human hepatocyte cancer HepG2 cells (Fig. 1C and Fig. 1D). These results demonstrated that MAVS was phosphorylated under physiological conditions of IFN-β activation. Interestingly, MAVS tyrosine phosphorylation triggered by viral infection began to decrease to a level lower than the uninfected cells beyond 120 min post-infection in HEK293T and MCF-7 cells, while MAVS tyrosine phosphorylation decreased to a level similar to the uninfected cells from 60 min after VSV infection in HepG2 cells, indicating that MAVS phosphorylation kinetics are cell type specific. Typically, HEK293T cells increased IFN-β production well over 24 h post-infection, the disappearance of positive signal below uninfected threshold in the early stages of viral infection suggests that other positive signals that control the activity of MAVS and its downstream signaling molecules during viral infection might exist.

### MAVS Y9F impairs MAVS-mediated innate immune signaling

**Figure 2:**
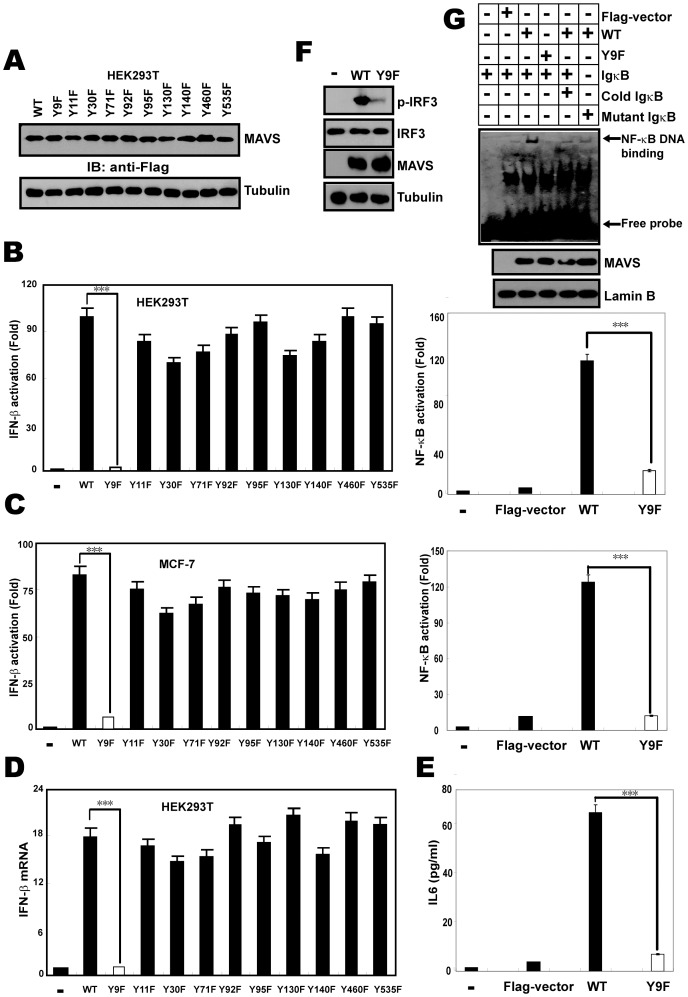
MAVS Y9F impairs MAVS-mediated innate immune signaling. (A) Expression of wild-type (WT) and mutant MAVS proteins was assessed by Western-blotting in HEK293T cells. (B–C) HEK293T cells (B) or MCF-7 cells (C) were transfected with expression vector encoding Flag-MAVS WT or Flag-MAVS mutants together with IFN-β-LUC or NF-κB-LUC. The LUC activity was measured 24 h later and normalized for transfection efficiency. Results are expressed as fold-increases of luciferase levels relatively to Flag-vector transfected cells. (D) HEK293T cells were transfected with a vector encoding Flag-MAVS or Flag-MAVS mutants and endogenous IFN-β mRNA expression was analyzed by qRTPCR. Results were expressed as fold-increase of IFN-β mRNA level normalized with β-actin level and relatively to Y9F MAVS-transfected cells. (E) Using the supernatants collected from the samples shown in panel B, IL6 release was measured by ELISA. From B to E, Data are mean of three independent experiments ± SEM done in triplicate.(F) HEK293T cells were transfected with expression vector encoding Flag-MAVS WT (WT) or Flag-MAVS Y9F mutant (Y9F). Whole cell lysates were analyzed by immunoblotting with anti-IRF3, anti-p-IRF3 or anti-Flag antibody, anti-Tubulin was used as equal loading control. (G) EMSA was performed using ^32^P-labeled consensus NF-κB probe and nuclear proteins extracted from HEK293T cells transfected with Flag-MAVS WT (WT) or Flag-MAVS Y9F mutant (Y9F).

In order to identify the relevant tyrosine residue(s) of MAVS required for its phosphorylation activity, all the tyrosine amino acids in human MAVS were substituted with phenylalanine residue individually. Plasmids encoding individually the 10 variants were generated by site-directed polymerase chain reaction (PCR) mutagenesis using a WT MAVS (accession number NM_020746) vector as a template. We first studied the impact of those mutations on the expression pattern of MAVS protein. To avoid a nonspecific cell-type effect, WT and YF mutational MAVS were expressed in two different human cell lines, the human embryonic kidney cell line HEK293T and human breast cancer cell line MCF-7. Western-blotting revealed that none of the YF mutations altered MAVS expression profile (Fig. 2A and data not shown). Next, we determined whether those YF mutations could alter MAVS-induced IFN-mediated antiviral and/or NF-κB-mediated pro-inflammatory signaling in the two foregoing cell types. In agreement with previous studies [3], [9], WT MAVS constitutively induced IFN-β as well as NF-κB responses relative to control cells (Fig. 2B and Fig. 2C). Interestingly, The Y9F mutation reduced IFN-β activation and NF-κB activation by nearly 95%, all the other MAVS mutants analyzed activated IFN-β to an extent similar to wild-type MAVS. The loss-of-function effect of Y9F MAVS variant was further confirmed by measuring the expression of endogenous inflammatory and antiviral mediators IFN-β and IL6 in HEK293T cells (Fig. 2D and Fig. 2E). Similarly, activation of NF-κB and IRF3 by EMSA and immunoblotting was found in HEK293T cells transfected with wild type MAVS, in contrast, expression of the MAVS Y9F mutant massively inhibited NF-κB-dependent transcription and IRF3 phosphorylation (Fig. 2F and Fig. 2G).

### MAVS Y9F affects TRAF3 and TRAF6 binding to MAVS

**Figure 3:**
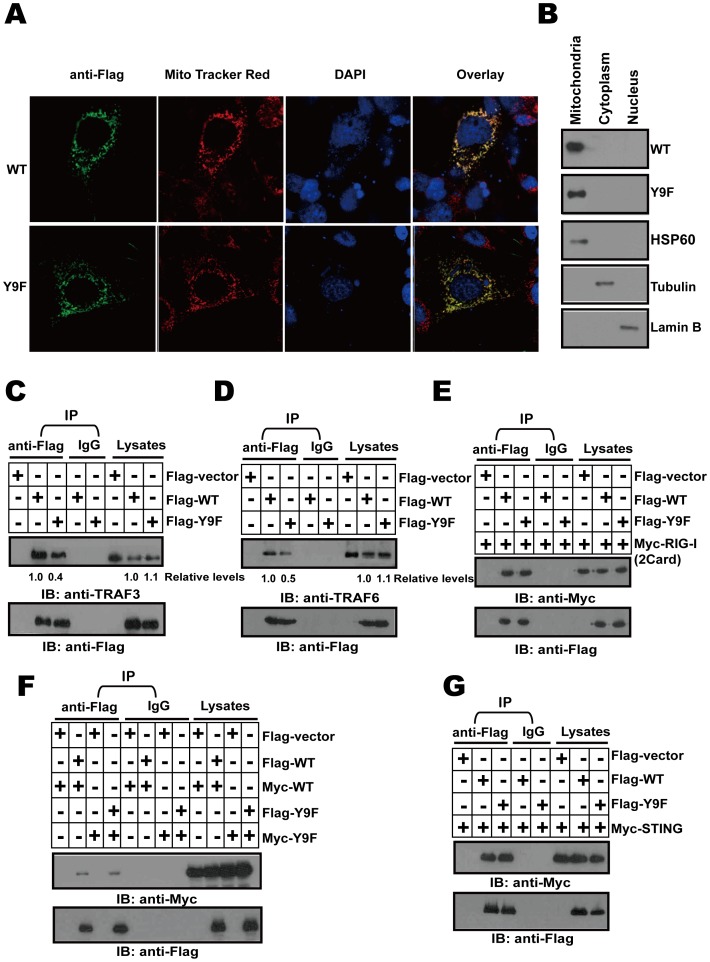
MAVS Y9F affects TRAF3 and TRAF6 binding to MAVS. (A) Hela cells were transfected with expression vector encoding Flag-MAVS WT (WT) or Flag-MAVS Y9F mutant (Y9F), cells were then stained with anti-Flag antibody and imaged by confocal microscopy. The mitochondria were stained with Mito Tracker. The yellow staining in the overlay image indicates co-localization of MAVS and Mito Tracker. (B) HEK293T cells were transfected with expression vector encoding Flag-MAVS (WT) or Flag-MAVS Y9F mutant (Y9F), mitochondria, cytoplasm and nucleus fractions were analyzed by immunoblotting with anti-Flag, anti-HSP60, anti-Lamin B and anti-Tubulin antibodies. (C–E and G) HEK293T cells were cotransfected with Flag-WT or Flag-Y9F together with Myc-tagged RIG-I 2Card (E) or Myc-tagged STING (G). After 48 h, cell lysates were immunoprecipitated with anti-Flag antibody, followed by immunoblotting with anti-Flag, anti-TRAF3 (C), anti-TRAF6 (D) and anti-Myc antibodies. (F) HEK293T cells were cotransfected with Flag-WT or Y9F and Myc-WT or Y9F. After 48 h, cell lysates were immunoprecipitated with anti-Flag antibody, followed by immunoblotting with anti-Flag (lower panel) and anti-Myc (upper panel) antibodies. From C–G, Each blot is a representative example of at least three independent experiments with similar results. Numbers below certain Western blots indicate relative levels determined by software-based quantitation of the representative experiment shown.

MAVS is a mitochondrial outer membrane protein, which is required for its self-association and IFN-β signaling. Thus we first analyzed the subcellular localization of MAVS Y9F mutant. We employed confocal microscopy to visualize Hela cells transfected with an expression vector encoding Flag-tagged MAVS or Flag-tagged MAVS Y9F mutant. Fluorescent immunostaining with a Flag-specific antibody showed a punctuate pattern of MAVS localization that overlapped with the staining pattern of Mito Tracker, a fluorescent marker taken up specifically by mitochondria. We also found by confocal microscopy that MAVS Y9F mutant was a mitochondria protein that overlapped with Mito Tracker staining just as WT MAVS (Fig. 3A).

To further determine if MAVS Y9F mutant is localized to the mitochondrial membrane, we carried out subcellular fractionation experiments. HEK293T cells were homogenized in an isotonic buffer that preserved mitochondria and other organelles, and the cell lysates were then subjected to differential centrifugation to separate cytosol and nucleus from mitochondria, MAVS Y9F mutant was found only in the mitochondria pellet, together with other mitochondrial proteins including HSP60 just as WT MAVS (Fig. 3B). Y9F MAVS and WT MAVS are both located in the mitochondria, suggesting that Y9F loss-of-function is neither due to protein degradation nor to a cellular localization error.

Next, we investigated the mechanism by which Y9F resulted in altering MAVS-mediated immune response. We hypothesized that such a mutation might alter MAVS conformation, thus modifying its interaction with downstream signaling molecules. Among them, TRAF3, TRAF6 and STING (stimulator of interferon genes) are critical MAVS signaling effectors [8], [16]. Interestingly, co-immunoprecipitation assays of overexpressed WT or Y9F with endogenous TRAF3 and TRAF6 demonstrated that the Y9F variant resulted in a significantly reduced interaction of MAVS with TRAF3 and TRAF6 (Fig. 3C and Fig. 3D). Remarkably, MAVS Y9F did not impair MAVS self-association (Fig. 3F) and its ability to interact with RIG-I 2 Cards and STING (Fig. 3E and Fig. 3G). These findings highlight a novel amino acid residue of MAVS that is crucial for the recruitment of TRAF3 but not for its self-association.

### MAVS Y9F alters MAVS-dependent antiviral responses to virus infection

**Figure 4:**
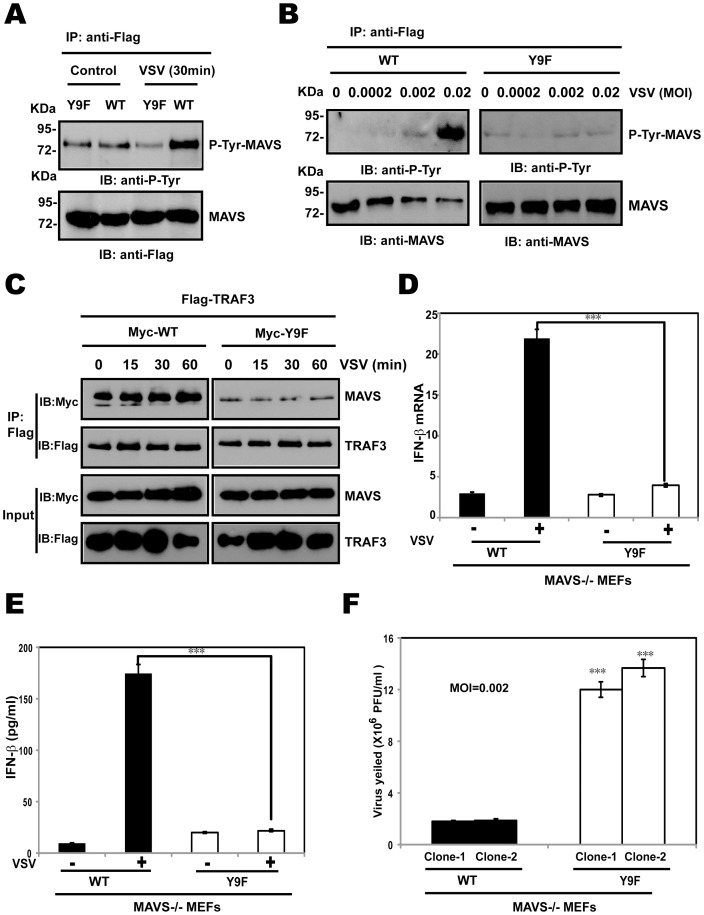
MAVS Y9F alters MAVS-dependent antiviral responses to virus infection. (A–B) HEK293T cells were transfected with expression vector encoding Flag-MAVS (WT) or Flag-MAVS Y9F mutant (Y9F). After 24 h, cells were infected with VSV at a MOI of 0.002 for 30 min (A) or at a different MOI (B) for 30 min. Anti-Flag immunoprecipitates were analyzed by immunoblotting with anti-P-Tyr or anti-MAVS antibody. (C) HEK293T cells were cotransfected with Myc-WT or Myc-Y9F together with Flag-tagged TRAF3 in the presence of VSV infection at a MOI of 0.002 for the indicated times, cell lysates were immunoprecipitated with anti-Flag antibody, followed by immunoblotting with anti-Flag and anti-Myc antibodies. (D) MAVS−/− MEF cells were transfected with expression vector encoding Flag-MAVS or Flag-MAVS Y9F mutant. After 24 h, cells were infected with VSV at a multiplicity of infection (MOI) of 0.002 for 20 h; endogenous IFN-β mRNA expression was analyzed by qRTPCR. Results are expressed as fold-increase of IFN-β mRNA level normalized with β-actin level and relatively to Y9F MAVS-transfected cells. (E) Using the supernatants collected from samples shown in panel D, IFN-β release was measured by ELISA. (F) Two individually transfected mixed clones with WT MAVS or Y9F MAVS in MAVS−/−MEFs were infected with VSV at a multiplicity of infection (MOI) of 0.002. Virus yields in the supernatants at 20 h after infection were determined by plaque assay. From D to F, Data are mean of three independent experiments ± SEM done in triplicate.

Since MAVS Y9 is a phosphorylation acceptor tyrosine that mediates TRAF3 and TRAF6 recruitment to MAVS, the ability of MAVS Y9F mutant to undergo phosphorylation was then examined. Phosphorylation was examined using a tyrosine-specific phosphorylation antibody with ectopically expressed wild-type MAVS or MAVS Y9F in HEK293T cells. A sharp reduction of tyrosine phosphorylation was observed in Y9F mutant compared to wild type MAVS upon VSV infection, indicating that tyrosine-9 of MAVS was the phosphorylation site (Fig. 4A and Fig. 4B). Both the WT MAVS and Y9F mutant were expressed well (Fig. 4A and Fig. 4B lower panel), thus the inability of MAVS Y9F mutant protein to induce phosphorylation is not due to ineffective protein expression. Importantly, co-immunoprecipitation assays of TRAF3 with WT or Y9F demonstrated that the TRAF3 could immunoprecipitate more WT MAVS than Y9F variant in response to VSV infection (Fig. 4C).

To confirm the relevance of the loss-of-function effect of Y9F MAVS in virus-infected cells, we examined the responsiveness of WT or Y9F MAVS to VSV viruses in MAVS−/− MEFs introduced by either WT MAVS or Y9F mutant. While WT MAVS rescue highly enhanced antiviral response triggered by viral infections, Y9F transfection dramatically impaired these responses. Remarkably, Y9F mutation resulted in complete loss-off induction on IFN-β mRNA and protein levels (Fig. 4D and Fig. 4E).

We next sought to determine whether MAVS Y9F mutant regulated the replication of VSV virus replication, as RIG-I-mediated IFN-β signaling is critical in restricting replication of this RNA virus. We therefore used VSV to infect MAVS−/− MEFs transfected with an expression vector encoding Flag-tagged MAVS or Flag-tagged MAVS Y9F mutant. MAVS Y9F mutant increased the production of VSV by about 6 fold (Fig. 4F), suggesting that MAVS phosphorylation at tyrosine −9 modulates the innate antiviral cellular response by acting as a positive regulator of RIG-I-mediated IFN-β signaling.

### MAVS Y9F is dispensable for MAVS-induced apoptosis

**Figure 5:**
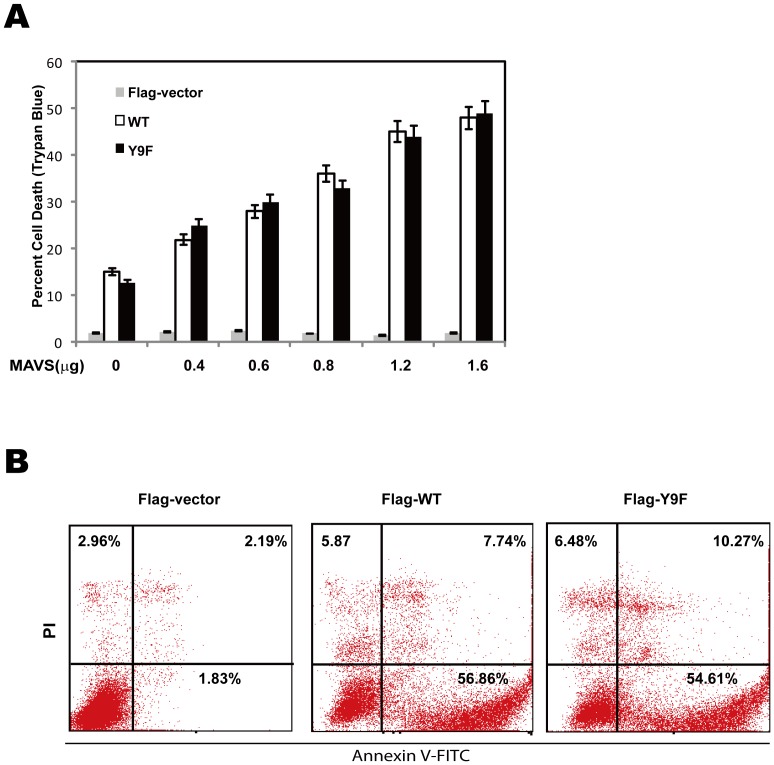
MAVS Y9F is dispensable for MAVS-induced apoptosis. (A) HEK293T cells were transfected with increasing amount of Flag-vector, Flag-WT or Flag-Y9F mutant. Cells were harvested for Trypan Blue counting 48 h post-transfection. Data are mean of three independent experiments ± SEM done in triplicate. (B) HEK293T cells were transfected with 1.0 µg expression vector encoding Flag-vector, Flag-WT or Flag-Y9F mutant. Annexin V assays were performed 48 hours post-transfection.

Previous reports have shown that the MAVS-induced caspase-dependent apoptosis and MAVS-induced apoptosis are independent of type-1 interferon production or NF-κB activity [17]. We had shown that MAVS tyrosine-9 site is required for IFN-β signaling, therefore we sought to determine the effect of Y9F mutation on MAVS-induced apoptosis. We expressed full length MAVS protein or MAVS Y9F mutant protein in HEK293T cells and measured trypan blue exclusion test of cell viability. In this assay, we observed a similar dose-dependent increase in the percentage of trypan blue positive cells at 48 hours posttransfection with WT MAVS or MAVS Y9F mutant (Fig. 5A). In consistence with this, the MAVS Y9F mutant, that impairs IFN-β signaling, was completely capable of inducing apoptosis just as MAVS WT (Fig. 5B), suggesting that, in contrast to the role of MAVS Y9 in interferon signaling, MAVS tyrosine-9 phosphorylation was dispensable for MAVS-induced apoptosis.

## Discussion

Ubiquitination and phosphorylation have emerged as key posttranslational modifications that control induction and shutdown of the interferon response [16], [18], [19]. The mechanism of activation of IKKε and TBK-1 by MAVS following RIG-I engagement involves formation of one or more multimeric complexes in which MAVS potentially interacts with multiple adapter molecules, such as TRAF2/3/6, TRADD (TNFRSF1A-associated via death domain), FADD (Fas -associated via death domain), NEMO, RIP-1 (receptor-interacting serine-threonine kinase 1), TANK (TRAF family member-associated NF-κB activator) and STING (stimulator of interferon genes) [5], [20], [21], [22]. Recent studies showed that ubiquitination at MAVS K500 mediates recruitment of IKKε to MAVS [5], [20], [21], [22]. Interestingly, IKKε recruitment to MAVS resulted in MAVS phosphorylation and negative regulation of the NF-κB pathway with a concomitant decrease in IFN-β and ISG (interferon stimulated gene) expressions [23]. However, the exact functions of MAVS tyrosine phosphorylation that regulate the IFN-β response are incompletely understood. In the present study, we demonstrated for the first time that MAVS undergoes extensive tyrosine phosphorylation following viral infection. More specifically, tyrosine-9 of MAVS was identified as a phosphorylation site that is required for IFN-β signaling. Indeed, MAVS Y9F mutation directly reduced TRAF3 and TRAF6 recruitment. Functionally, MAVS tyrosine-9 phosphorylation contributed to MAVS antiviral function without interfering with its apoptosis property.

A recent report demonstrated that the transmembrane domain is essential for MAVS to both self-associate and signal [24], [25]. Self-association of the MAVS N-terminal CARD allows for the direct binding and activation of TRAF3 [20], [26]. Our reports showed that MAVS Y9F mutation did not change its mitochondria localization and self-association property, MAVS Y9F also showed intact card-card domain interaction with RIG-I. However, MAVS Y9F mutant protein displayed reduced interaction with TRAF3/6 and impaired its antiviral function, therefore, phosphorylation of MAVS at tyrosine-9 modulates the innate antiviral cellular response through recruitment of TRAF3/6 by acting as a positive regulator of RIG-I-mediated IFN-β signaling.

In addition to K63-ubiquitination at MAVS K500, MAVS can link K48-polyubiquitin chains following virus infection and is the target for proteasomal degradation by the E3 ligase RNF125, suggesting that MAVS may undergo multiple ubiquitination events. Ning *et al.* recently demonstrated that K63-linked ubiquitination of C-terminal residues of IRF7 occurred as a prelude to IRF7 phosphorylation and activation [27]. It is thus quite possible that MAVS ubiquitination is required for its phosphorylation. Paz *et al* argued that IKKε directly phosphorylated MAVS and that this serine or threonine phosphorylation modification of MAVS played a negative control in IFN-β signaling [23]. Specifically, our evidence suggests that MAVS tyrosine phosphorylation triggered by viral infection plays positive regulatory role in the MAVS signaling pathway. As protein phosphorylation can lead to activation or inactivation of the substrate, depending on its nature. Further studies are needed to address the exact relationship between ubiquitination, phosphorylation and phosphorylation type of MAVS and to delineate MAVS upstream kinases.

It is known that MAVS is a potent inducer of IFN-I responses and that IFN-I can activate host apoptotic responses, therefore it was important to determine whether the cell death response observed in the current study was a consequence of IFN-I secretion. Lei *et al* demonstrated that MAVS-mediated apoptosis was not a consequence of IFN-I induction by MAVS [17]. Our results showing MAVS Y9F mutant ablated IFN-β production with intact apoptosis property supported a duality of function for the MAVS protein in regulating both IFN-I and apoptotic antiviral responses from within the mitochondria, suggesting that MAVS is a pivotal molecule in the bifurcation of host responses following viral challenge.

In summary, here we have demonstrated that phosphorylation modification is involved in the control of MAVS activity in innate immune response, which thereby provides positive regulation of the innate antiviral response against infection by RNA viruses. Phosphorylation at tyrosine-9 is essential events to regulate the activity of MAVS. Uncovering the mechanism of MAVS phosphorylation state controlled by protein kinases and phosphatases will help us to further understand the function of MAVS at the molecular level.
